# Relative frequency of inherited retinal dystrophies in Brazil

**DOI:** 10.1038/s41598-018-34380-0

**Published:** 2018-10-29

**Authors:** Fabiana Louise Motta, Renan Paulo Martin, Rafael Filippelli-Silva, Mariana Vallim Salles, Juliana Maria Ferraz Sallum

**Affiliations:** 1 Department of Ophthalmology, Universidade Federal de São Paulo, São Paulo, Brazil; 2 Department of Biophysics, Universidade Federal de São Paulo, São Paulo, Brazil; 3 Institute of Genetic Medicine, Johns Hopkins Medicine, Baltimore, USA; 4 Instituto de Genética Ocular, Sao Paulo, Brazil

## Abstract

Among the Brazilian population, the frequency rates of inherited retinal dystrophies and their causative genes are underreported. To increase the knowledge about these dystrophies in our population, we retrospectively studied the medical records of 1,246 Brazilian patients with hereditary retinopathies during 20 years of specialized outpatient clinic care. Of these patients, 559 had undergone at least one genetic test. In this cohort, the most prevalent dystrophies were non-syndromic retinitis pigmentosa (35%), Stargardt disease (21%), Leber congenital amaurosis (9%), and syndromic inherited retinal dystrophies (12%). Most patients had never undergone genetic testing (55%), and among the individuals with molecular test results, 28.4% had negative or inconclusive results compared to 71.6% with a conclusive molecular diagnosis. *ABCA4* was the most frequent disease-causing gene, accounting for 20% of the positive cases. Pathogenic variants also occurred frequently in the *CEP290*, *USH2A*, *CRB1*, *RPGR*, and *CHM* genes. The relative frequency rates of different inherited retinal dystrophies in Brazil are similar to those found globally. Although mutations in more than 250 genes lead to hereditary retinopathies, only 66 genes were responsible for 70% of the cases, which indicated that smaller and cheaper gene panels can be just as effective and provide more affordable solutions for implementation by the Brazilian public health system.

## Introduction

It is estimated that 253 million people are visually impaired worldwide; the majority have moderate to severe visual impairment and 36 million are blind^1^. Among children under 15 years of age, approximately 14 million are blind globally^2^. In high- and middle-income countries, the most common causes of childhood blindness are cerebral visual impairment, optic nerve hypoplasia, and inherited retinal disorders^3^.

Inherited retinal dystrophies (IRDs) constitute a group of diseases with vast clinical and genetic heterogeneity that affect about 1 in 2,000 to 3,000 individuals^4^. According to the Retinal Information Network^5^, more than 250 genes already have been implicated as causes of retinal diseases, many of which may cause more than one phenotype. The IRD features are related primarily to progressive retinal degeneration with significant decreases in or total loss of vision. The disease onset, progression, and severity vary and are difficult to predict. The modes of inheritance can range from autosomal dominant and recessive to X-linked^6^.

The most common forms of IRDs are retinitis pigmentosa (RP), a rod-cone dystrophy that affects about 1 in ~4,000 individuals^7^ and can result from mutations in approximately 90 genes^5^, among them the *ABCA4*, *BEST1*, *CERKL*, *CRB1*, *CRX*, *EYS*, *IFT140*, *MERTK*, *PDE6B*, *RHO*, *RP1*, *RP1L1*, *RP2*, *RPE65*, *RPGR*, and *USH2A* genes; Stargardt disease (STGD), a type of macular dystrophy with a prevalence of 1 in 10,000 individuals^8^, that usually is caused by pathogenic variants in the *ABCA4*^9,10^, *ELOV4*^11^, or *PROM1*^12^ genes; and Usher syndrome, the most frequent syndromic RP affecting 1 in 20,000 Caucasian individuals^13^, that has deafness as its main feature in association with visual impairment^14,15^ due to mutations in the *ABHD12*, *ADGRV1*, *ARSG*, *CDH23*, *CEP250*, *CEP78*, *CIB2*, *CLRN1*, *DFNB31*, *HARS*, *MYO7A*, *PCDH15*, *USH1C*, *USH1G*, and *USH2A* genes^5^.

In Brazil, about 506,000 people are blind, of whom approximately 66,000 (13%) are children^16^. In 2012, the Brazilian Council of Ophthalmology estimated that about 50,000 people have RP^17^. In the Brazilian population, the frequency rate of IRDs and their causative genes are underreported. Our group published the only study that showed the relative frequency of retinopathies in southeastern Brazil^18^, with the results based only on the clinical diagnoses of 93 patients.

The current study is the result of 20 years of care of patients with IRDs that describes the relative frequencies of different diseases, molecular diagnostic success rates, and disease-causing genes in the Brazilian population.

## Results

### Clinical diagnosis

**Figure 1:**
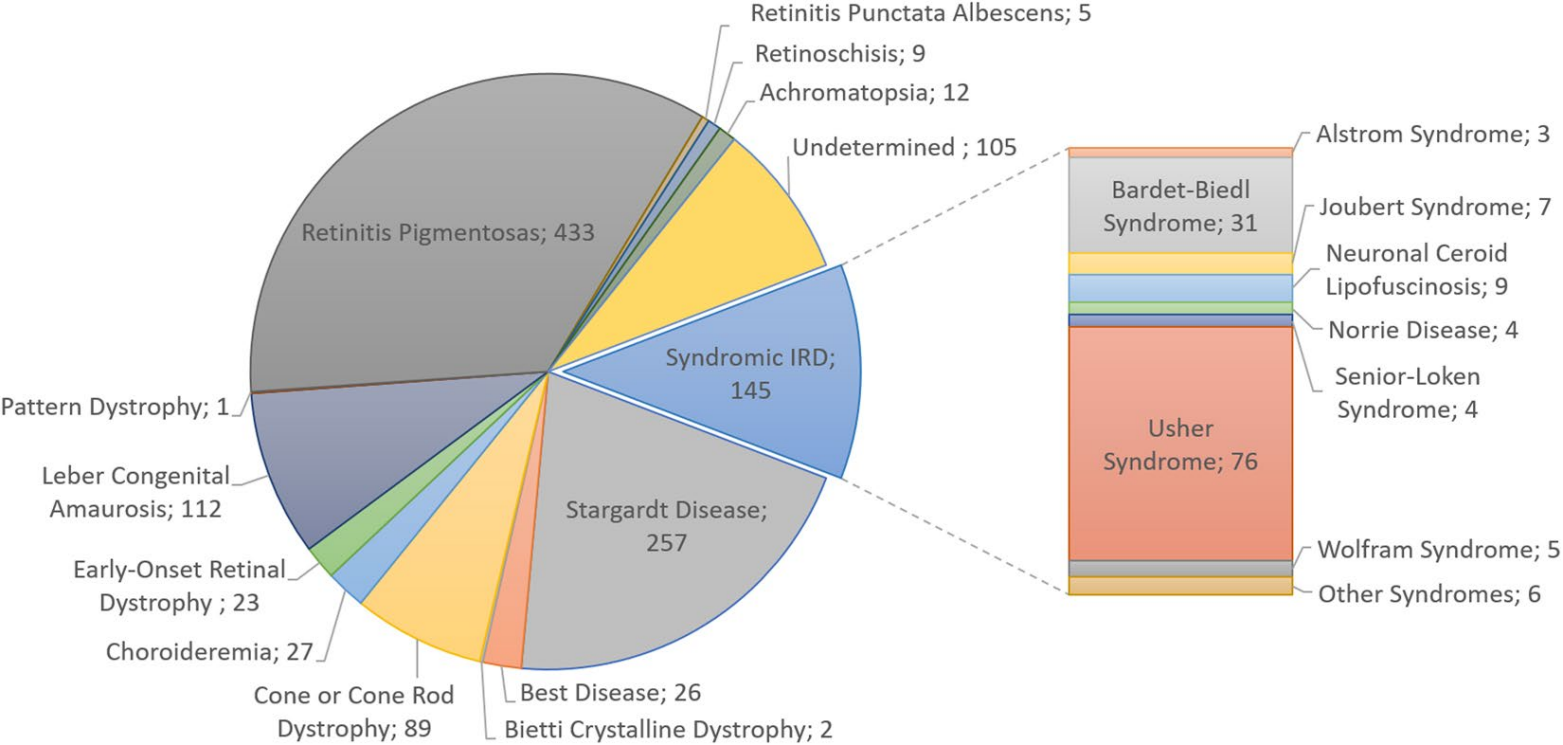
The distribution of patients according to their clinical diagnoses (n = 1,246). The syndromic IRD cases are grouped in the pie chart, and detailed in the stacked bar on the right. Arts syndrome, Hallervorden-Spatz disease, Jalili syndrome, Marshall syndrome, Microcephaly and chorioretinopathy and PHARC syndrome (Polyneuropathy, Hearing loss, Ataxia, Retinitis pigmentosa, and Cataract) are designated as “other syndromes”.

Over the previous 20 years, 1,246 patients with retinopathy who were members of 1,159 different families have been evaluated. Among the individuals, 906 were sporadic cases, 161 were related patients, and 179 had other affected relatives who were not included in this study. In addition, 122 (>10%) families had the feature of consanguinity. Most patients had non-syndromic RP (~35%), followed by patients with macular impairment caused by STGD (~21%) and patients with severe congenital and early-onset IRDs that represented approximately one-tenth of all cases (Leber congenital amaurosis [LCA], 9% and early-onset retinal dystrophy [EORD], ~2%) (Fig. 1). The syndromic forms of retinal dystrophies accounted for 12% of all patients in this study, wherein ciliopathies were more prominent, affecting 9.6% of the cases (Usher syndrome 6%, Bardet-Biedl syndrome 2.5%, Joubert syndrome 0.56%, Senior-Løken syndrome 0.32%, and Alström syndrome 0.24%) (Fig. 1). Among the metabolic disorders, neuronal ceroid lipofuscinosis was the most representative (0.72%). In this study, choroideremia was the only disease found among the chorioretinal dystrophies (2.2%). Some rare syndromes also were observed at a frequency of 0.08%, i.e., Arts syndrome, Hallervorden-Spatz disease, Jalili syndrome, Marshall syndrome, Microcephaly and chorioretinopathy, and Polyneuropathy, Hearing loss, Ataxia, Retinitis pigmentosa, and Cataract syndrome (PHARC) (Fig. 1).

A clinical diagnosis was not established in 105 (8.4% patients (Fig. 1), only 23 of whom had undergone genetic testing; however, the results were either negative or inconclusive. Therefore, molecular tests were not helpful in diagnosing those 23 cases.

### Types of molecular tests

In this cohort, 687 (55%) patients have never undergone genetic testing. In another 559 individuals from 514 families, different commercial molecular tests were performed, of which some were more specific and others broader. DNA array testing (Arrayed Primer Extension [APEX]) was used to detect known variants, and in one case, microarray-based Comparative Genomic Hybridization (aCGH) was performed to detect copy number variations. The sequencing techniques predominated, such as Sanger sequencing and Next Generation Sequencing (NGS) (gene panels or whole exome). Among those patients with molecular test results, 159 (28.4%) had negative or inconclusive results (Table 1).

**Table 1:** Number of patients according to the sort of molecular test performed.

Molecular Test	Total Patients	Total Positive Results (%)^*^
aCGH	1	1 (100%)
APEX	52	14 (26.9%)
Exome	9	6 (66.7%)
NGS panels	453	348 (76.8%)
Sanger Sequencing	44	31 (70.5%)

The total number of patients that underwent molecular testing is 559, with positive results in 400.

^*^Percentage based on the total number of patients who underwent molecular testing.

aCGH: microarray comparative genomic hybridization; APEX: arrayed primer extension; NGS panels: next-generation sequencing gene panel.

The NGS gene panels, technology that was used most often, represented 81% of all genetic testing and established a diagnosis in 77% of cases (348 of 453 patients) (Table 1). On the other hand, genotyping using APEX resulted in 83% of the negative or inconclusive cases (38 of 52 patients). More robust and broader genetic tests, such as whole exome sequencing (WES), were sparsely used. Only nine patients underwent WES and six of them (67%) received conclusive results (Table 1). Sanger sequencing was performed in 44 individuals, and achieved definitive diagnoses in about 70.5% of the cases. Only one gene was analyzed in 84% of the Sanger sequencing cases.

### IRD Genetic Findings

**Figure 2:**
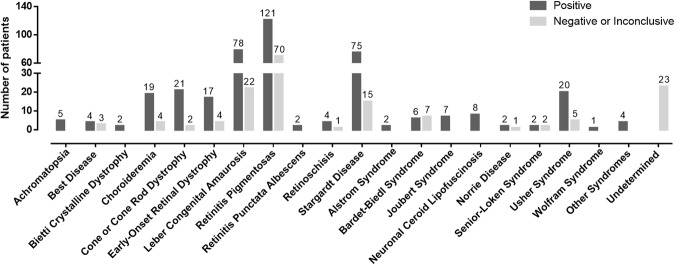
The distribution of positive and negative/inconclusive tests according to the IRD type (n = 559; 400 positive and 159 negative/inconclusive results).

The overall phenotype distribution according to the test result is shown on Fig. 2, whereas Tables 2 and 3 show the details of the genetic findings. The disorders with the lowest success rates were the Bardet-Biedl and Senior-Løken syndromes, with conclusive results found in half or fewer of the cases. In contrast, achromatopsia, Bietti crystalline dystrophy, retinitis punctata albescens, Alström syndrome, Joubert syndrome, neuronal ceroid lipofuscinosis, and Wolfram syndrome had disease-causing genes detected in all tested cases. The positive results of the most frequent phenotypes (RP, STGD, LCA, Usher syndrome, cone or cone-rod dystrophy, choroideremia, and EORD) accounted for 74% of the cases.

**Table 2:** Number of affected patients with positive genetic testing per retinopathy.

IRD	Affected Patients	Disease-causing genes (n - inheritance pattern)
Achromatopsia	5	*CNGA3* (*1-*AR*)*, *CNGB3* (*3-*AR*)*, *PDE6C* (*1-*AR)
Best disease	4	***BEST1*** (*3-*AD, AR), *PRPH2* (*1-*AD)
Bietti crystalline dystrophy	2	*CYP4V2* (*1-*AR), *ITM2B* (*1-*AD)
Choroideremia	19	***CHM*** (*19-*XL)
Cone or Cone-Rod Dystrophy	21	***ABCA4*** (*11-*AR), *CERKL* (*3-*AR), *CNGA3* (*1-*AR), *GUCA1A* (*1-*AD), *OPN1LW/OPN1WM* (*1-*XL), *PROM1* (*1-*AR), *PRPH2* (*2-*AD), *RPGR* (*1-*XL)
Early-onset retinal dystrophy	17	***ABCA4*** (*4-*AR), *CNGB1* (*1-*AR), *CRB1* (*3-*AR), *IQCB1* (*1-*AR), *PDE6B* (*1-*AR), *PROM1* (*2-*AR), *PRPH2* (*1-*AD), *RDH12* (*1-*AR), *RP1* (*2-*AR), *TULP1* (*1-*AR)
Leber congenital amaurosis	78	***CEP290*** (*20-*AR), *CRB1* (*12-*AR), *CRX* (*1-*AD), *GUCY2D* (*5-*AR), *IQCB1* (*2-*AR), *LCA5* (*2-*AR), *LRAT* (*3-*AR), *NMNAT1* (*5-*AR), *NPHP4* (*1-*AR), *RDH12* (*4-*AR), *RPE65* (*16-*AR), *RPGRIP1* (*6-*AR), *SPATA7* (*7-*AR)
Retinitis pigmentosa	121	*BBS1* (*1-*AR), *BBS2* (*1-*AR), *CDHR1* (*1-*AR), *CERKL* (*7-*AR), *CNGA1* (*1-*AR), *CNGB1* (*3-*AR), *CRB1* (*5-*AR), *CRX* (*1-*AD), *EYS* (*16-*AR), *GPR98†* (*1-*AR), *HGSNAT* (*2-*AR), *MERTK* (*6-*AR), *MKS1* (*1-*AR), *NR2E3* (*2-*AD), *PDE6A* (*2-*AR), *PDE6B* (*5-*AD, AR), *PROM1* (*2-*AR), *PRPF31* (*6-*AD), *PRPF8* (*3-*AD), *PRPH2* (*1-*AR), *RDH12* (*1-*AR), *RHO* (*6-*AD), *RIMS1* (*1-*AD), *ROM1* (*1-*AD), *RP1* (*7-*AD, AR), *RP2* (*2-*XL), ***RPGR*** (*19-*XL), *SNRNP200* (*4-*AD), *TULP1* (*1-*AR), *USH2A* (*11-*AR), *WDR19* (*1-*AR)
Retinitis punctata albescens	2	***RLBP1*** (*2-*AD)
Retinoschisis	4	***COL2A1*** (*2-*AD), ***RS1*** (*2-*XL)
Stargardt disease	75	***ABCA4*** (*69-*AR), *PROM1* (*4-*AD, AR), *PRPH2* (*2-*AD)
**Syndromic IRD**		
Alström syndrome	2	***ALMS1*** (*2-*AR)
Arts syndrome	1	***PRPS1*** (*1-*XL)
Bardet-Biedl syndrome	6	***BBS1*** (*6-*AD)
Jalili syndrome	1	***CNNM4*** (*1-*AR)
Joubert syndrome	7	***AHI1*** (*4-*AR), *CEP290* (*2-*AR), *INPP5E* (*1-*AR)
Microcephaly and chorioretinopathy	1	***TUBGCP4*** (*1-*AR)
Neuronal ceroid lipofuscinosis	8	***CLN3*** (*8-*AR)
Norrie disease	2	***NDP*** (*2-*XL)
PHARC* syndrome	1	***ABHD12*** (*1-*AR)
Senior-Løken syndrome	2	***IQCB1*** (*1-*AR), ***NPHP4*** (*1-*AR)
Usher syndrome	20	*ABHD12* (*1-*AR), *CLRN1* (*1-*AR), *MYO7A* (*8-*AR), ***USH2A*** (*10-*AR)
Wolfram syndrome	1	***WFS1*** (*1-*AD)

The boldface type indicates the most frequent disease-causing genes.

The total number of patients affected by non-syndromic IRD is 348 and by syndromic IRD 52.

AD: autosomal dominant; AR: autosomal recessive; XL: X-linked inheritance.

^*^PHARC: Polyneuropathy, Hearing loss, Ataxia, Retinitis pigmentosa and Cataract.

^†^Also known as ADGRV1.

**Table 3:** Number of affected patients with positive genetic testing per gene.

Gene	Affected Patients		Retinal Dystrophy (n)
	n	%^†^	
*ABCA4*	84	21.0	Cone or cone-rod dystrophy (11), early-onset retinal dystrophy (4), Stargardt disease (69)
*CEP290*	22	5.50	Joubert syndrome (2), Leber congenital amaurosis (20)
*USH2A*	21	5.25	Retinitis pigmentosa (11), Usher syndrome (10)
*CRB1*	20	5.00	Early-onset retinal dystrophy (3), Leber congenital amaurosis (12), retinitis pigmentosa (5)
*RPGR*	20	5.00	Cone or cone-rod dystrophy (1), retinitis pigmentosa (19)
*CHM*	19	4.75	Choroideremia (19)
*EYS*	16	4.00	Retinitis pigmentosa (16)
*RPE65*	16	4.00	Leber congenital amaurosis (16)
*CERKL*	10	2.50	Cone or cone-rod dystrophy (3), retinitis pigmentosa (7)
*PROM1*	9	2.25	Cone or cone-rod dystrophy (1), early-onset retinal dystrophy (2), retinitis pigmentosa (2), Stargardt disease (4)
*RP1*	9	2.25	Early-onset retinal dystrophy (2), retinitis pigmentosa (7)
*CLN3*	8	2.00	Neuronal ceroid lipofuscinosis (8)
*MYO7A*	8	2.00	Usher syndrome (8)
*BBS1*	7	1.75	Bardet-Biedl syndrome (6), retinitis pigmentosa (1)
*PRPH2*	7	1.75	Best disease (1), cone or cone-rod dystrophy (2), early-onset retinal dystrophy (1), retinitis pigmentosa (1), Stargardt disease (2)
*PRPF31*	6	1.50	Retinitis pigmentosa (6)
*MERTK*	6	1.50	Retinitis pigmentosa (6)
*PDE6B*	6	1.50	Early-onset retinal dystrophy (1), retinitis pigmentosa (5)
*RDH12*	6	1.50	Early-onset retinal dystrophy (1), Leber congenital amaurosis (4), retinitis pigmentosa (1)
*RHO*	6	1.50	Retinitis pigmentosa (6)
*RPGRIP1*	6	1.50	Leber congenital amaurosis (6)
*GUCY2D*	5	1.25	Leber congenital amaurosis (5)
*NMNAT1*	5	1.25	Leber congenital amaurosis (5)
*AHI1*	4	1.00	Joubert Syndrome (4)
*CNGB1*	4	1.00	Early-onset retinal dystrophy (1), retinitis pigmentosa (3)
*IQCB1*	4	1.00	Early-onset retinal dystrophy (1), Leber congenital amaurosis (2), Senior-Løken syndrome (1)
*SNRNP200*	4	1.00	Retinitis pigmentosa (4)
*BEST1*	3	0.75	Best disease (3)
*CNGB3*	3	0.75	Achromatopsia (3)
*LRAT*	3	0.75	Leber congenital amaurosis (3)
*PRPF8*	3	0.75	Retinitis pigmentosa (3)
*ABHD12*	2	0.50	PHARC* syndrome (1), Usher syndrome (1)
*ALMS1*	2	0.50	Alstrom syndrome (2)
*CNGA3*	2	0.50	Achromatopsia (1), cone or cone-rod dystrophy (1)
*COL2A1*	2	0.50	Retinoschisis (2)
*CRX*	2	0.50	Leber congenital amaurosis (1), retinitis pigmentosa (1)
*HGSNAT*	2	0.50	Retinitis pigmentosa (2)
*LCA5*	2	0.50	Leber congenital amaurosis (2)
*NDP*	2	0.50	Norrie disease (2)
*NPHP4*	2	0.50	Leber congenital amaurosis (1), Senior-Løken syndrome (1)
*NR2E3*	2	0.50	Retinitis pigmentosa (2)
*PDE6A*	2	0.50	Retinitis pigmentosa (2)
*RLBP1*	2	0.50	Retinite punctata albescens (2)
*RP2*	2	0.50	Retinitis pigmentosa (2)
*RS1*	2	0.50	Retinoschisis (2)
*TULP1*	2	0.50	Early-onset retinal dystrophy (1), retinitis pigmentosa (1)
*BBS2*	1	0.25	Retinitis pigmentosa (1)
*CDHR1*	1	0.25	Retinitis pigmentosa (1)
*CLRN1*	1	0.25	Usher syndrome (1)
*CNGA1*	1	0.25	Retinitis pigmentosa (1)
*CNNM4*	1	0.25	Jalili syndrome (1)
*CYP4V2*	1	0.25	Bietti crystalline dystrophy (1)
*GPR98* (*ADGRV1)*	1	0.25	Retinitis pigmentosa (1)
*GUCA1A*	1	0.25	Cone or Cone-Rod Dystrophy (1)
*INPP5E*	1	0.25	Joubert syndrome (1)
*ITM2B*	1	0.25	Bietti crystalline dystrophy (1)
*MKS1*	1	0.25	Retinitis pigmentosa (1)
*OPN1LW/OPN1WM*	1	0.25	Cone or Cone-Rod Dystrophy (1)
*PDE6C*	1	0.25	Achromatopsia (1)
*PRPS1*	1	0.25	Arts syndrome (1)
*RIMS1*	1	0.25	Retinitis pigmentosa (1)
*ROM1*	1	0.25	Retinitis pigmentosa (1)
*SPATA7*	1	0.25	Leber congenital amaurosis (1)
*TUBGCP4*	1	0.25	Microcephaly and chorioretinopathy (1)
*WDR19*	1	0.25	Retinitis pigmentosa (1)
*WFS1*	1	0.25	Wolfram syndrome (1)

Total of disease-causing genes is 66.

*PHARC: Polyneuropathy, Hearing loss, Ataxia, Retinitis pigmentosa and Cataract.

^†^Percentage based on the total number of patients with positive genetic test (n = 400).

Non-syndromic RP, the most common phenotype in this cohort, was diagnosed conclusively in six of ten cases (121 of 191 cases were positive). Pathogenic variants in 31 different genes led to this retinopathy, 18 of which caused only non-syndromic RP. The most mutated gene in this group was *RPGR* (19 patients), which causes an X-linked form of RP, followed by the *EYS* and *USH2A* genes, which caused 16 and 11 autosomal recessive cases, respectively. The latter was also responsible for half of the Usher syndrome cases (10 of 20 positive cases of this syndrome). This autosomal recessive syndrome had a success rate of 80% (20 conclusive cases of the 25 tested cases). Besides the *USH2A* gene, Usher syndrome also was caused by *ABHD12* (1; 5%), *CLRN1* (1; 5%), and *MYO7A* (8; 40%) genes.

Among the childhood retinal dystrophies, LCA had 78% of positive results, with 13 different disease-causing genes. Together, *CEP290* (20), *RPE65* (16), and *CRB1* (12) accounted for 61.5% of the conclusive results in this retinopathy. *CRB1* mutations also led to EORD (3 patients). The latter disease also was caused by nine other genes, including the *ABCA4*, which presented with a similar frequency (4 individuals) to the *CRB1* gene. In the current study, molecular testing identified eight of ten cases of EORD. The *ABCA4* gene also was the most commonly mutated gene in cone or cone-rod dystrophy and STGD, accounting for 50% and 90% of the conclusive tests, respectively.

**Figure 3:**
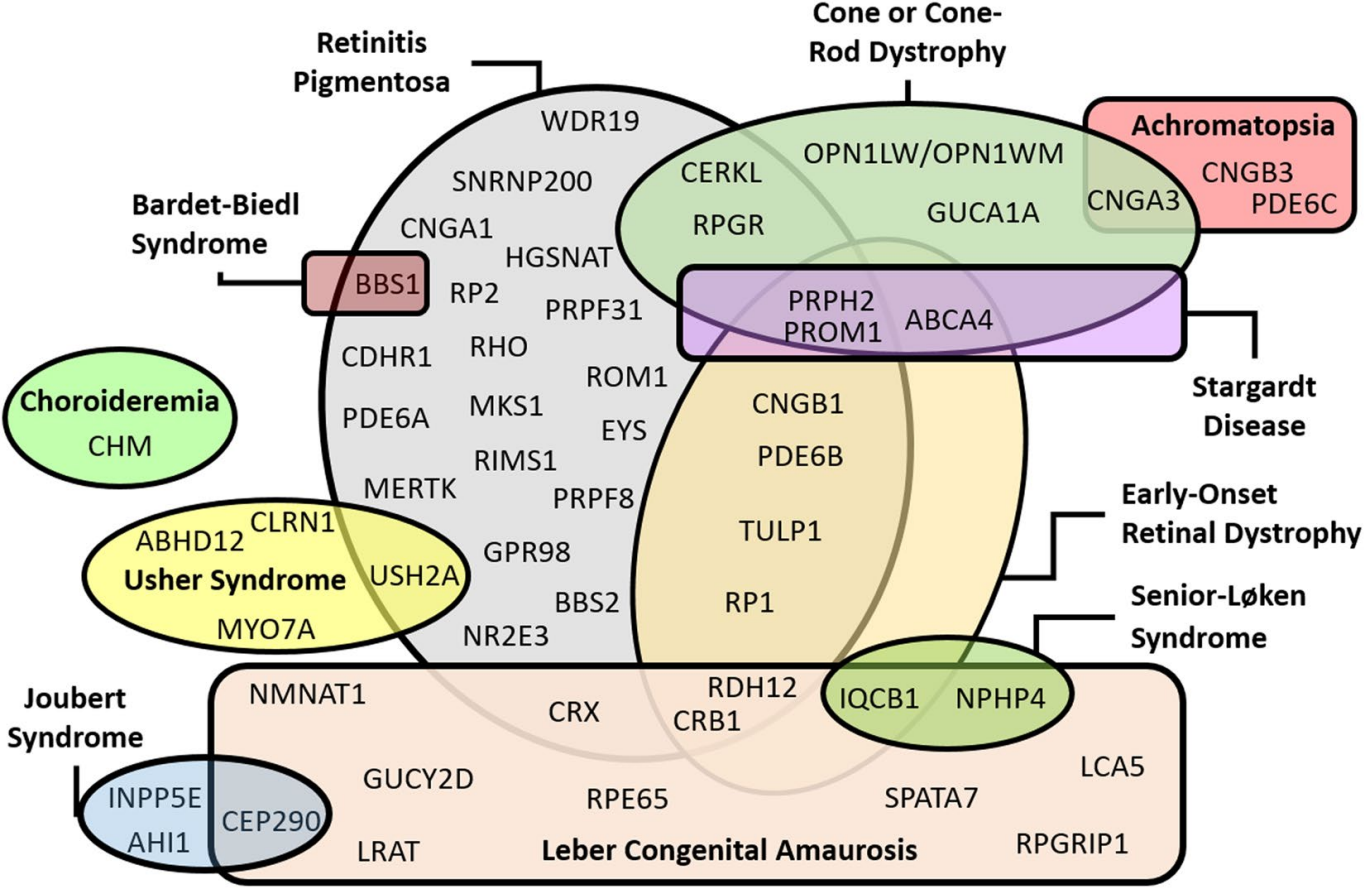
The diagram summarizes the genetic findings in the Brazilian sample and highlights the genetic overlap among some retinopathies.

IRDs are characterized by genetic heterogeneity, in which several genes can cause a single disease, whereas one gene can cause more than one type of retinopathy. Figure 3 illustrates this intrinsic feature and summarizes some of the current findings. Genes such as *PRPH2* and *PROM1* caused five and four different types of retinopathies, respectively, with predominant autosomal dominant traits for the *PRPH2* gene and autosomal recessive traits for the *PROM1* gene (Table 2). However, genes that cause only one type of IRD, such as the *EYS* (recessive RP25), *MYO7A* (recessive Usher syndrome type 1B), and *CLN3* (recessive neuronal ceroid lipofuscinosis 3) also were found in this patient cohort. Regarding the *CLN3* gene, deletions larger than 1 Kb were the predominant pathogenic variants.

In the current study, IRD-related genetic mutations were found in 66 different genes (Table 3), accounting for 71.6% of analyzed cases. The *ABCA4* gene was the most frequent disease-causing gene, representing 20% of the positive cases. Approximately four of ten patients with *ABCA4* mutations had at least one complex allele, i.e., two pathogenic variants in cis-compound heterozygosity (same allele). Other more representative genes were *CEP290*, *USH2A*, and *CRB1* that caused autosomal recessive diseases and *RPGR* and *CHM* that caused X-linked IRD; each one accounted for about 5% of the conclusive results.

*CEP290* mutations were found in 20 patients with LCA and two patients with Joubert syndrome. The latter exhibited only canonical splice site variants and nonsense or frameshift changes, while half of the patients with LCA had at least one c.2991 + 1655A > G variant. Premature stop codon formations were common in patients with mutations in the *USH2A* gene (85%). In addition, half of the cases with Usher syndrome caused by *USH2A* mutations had two different premature stop codon formations in each allele (trans-compound heterozygous).

The *CRB1* gene was mutated in 20 patients, causing RP (5), LCA (12), and EORD (3). Nine of ten alleles had missense variants in patients with RP, while cases with childhood retinal dystrophy had approximately 87% of alleles with a premature stop codon formation or changes in the Cys948 amino acid.

As mentioned previously, the *RPGR* gene was the most common cause of RP in this sample, and six of ten cases had a pathogenic variant in the ORF15 region. The *CHM* gene caused only choroideremia, an X-linked form of IRD, and about 60% of patients with a mutation in the *CHM* gene had large deletions ranging from loss of two exons to deletion of the entire coding region.

The six most commonly mutated genes (*ABCA4*, *CEP290*, *USH2A*, *CRB1*, *RPGR*, and *CHM*) in Brazilian patients with IRDs accounted for 46.5% of the positive results and 33.2% of the molecularly analyzed cases.

## Discussion

The remarkable genetic heterogeneity of the IRDs is due not only to the large number of involved genes but also to the fact that mutations in a specific gene can cause distinct phenotypes that vary widely in severity, progression, and mode of inheritance^19^. The current results showed clearly that mutations in some genes cause different retinopathies, such as the *CRB1* gene that causes EORD, LCA, and RP and the *ABCA4* gene that causes cone or cone-rod dystrophy, EORD, and STGD. In addition, diseases with different inheritance patterns can result from one gene, e.g., the *PROM1* gene, which leads to autosomal dominant STGD or autosomal recessive forms of RP, EORD, and cone or cone-rod dystrophy.

Moreover, some genes can cause both syndromic and non-syndromic forms of retinal dystrophies. In the current study, the *BBS1*, *CEP290*, *IQCB1*, *NPHP4*, and *USH2A* genes exhibited this characteristic. From a disease perspective, 12 of 23 phenotypes had more than one disease-causing gene, e.g., non-syndromic RP was caused by 31 genes, LCA by 13 different genes, and syndromic forms such as Usher and Joubert syndromes by four and three disease-causing genes, respectively.

The current findings agree with those of other studies and show the predominance of RP (~40%) in this population, with the syndromic RP form accounting for 20% of the RP cases^20–22^. Mutations in more than 80 genes already have been ascribed as causes of non-syndromic RP^5^ worldwide, while in this sample of Brazilian population, we report 31 different disease-causing genes. In addition, the most common cause of RP was mutations in the *RPGR* gene, more specifically in the OFR15 region^23,24^. Usher syndrome was also the most common type of syndromic RP^13,20^ in the current study, accounting for about 14% of patients with RP. Although more than ten genes can cause this disorder^5^, only four were responsible for Usher syndrome in this Brazilian sample. The overwhelming majority of patients had pathogenic variants in the *USH2A* and *MYO7A* genes (90% of Usher cases)^21,25^.

LCA accounts for 5% of retinal dystrophies^26,27^, with the *CEP290* gene likely being the most common mutated gene with the recurrent pathogenic variant the c.2991 + 1655A > G^27,28^, which corroborated our data. The *RPE65* gene causes 3% to 16% of LCA^26,27^ and has already been associated with RP20^29^. In the current study, this gene was responsible for 16 of 78 LCA-positive cases, whereas no patients with RP had the *RPE65* mutation. In 15% of patients with LCA, *CRB1* was the disease-causing gene and showed the same pattern between the pathogenic variant type and the severity of disease observed in other studies^30,31^. LCA is predominantly an autosomal recessive disease; however, autosomal dominant cases have already been reported^5,32^. In the current study, only one patient with the *CRX* gene mutation had this dominant trait.

Among the maculopathies, STGD was the predominant disease, with *ABCA4* mutations the most common. About one-third of patients with pathogenic variants in the *ABCA4* gene had complex alleles^33^, which reinforce the need for familiar segregation analysis to determine if both alleles have at least one pathogenic variant. In the current study, the *PROM1* gene caused STGD with autosomal dominant and recessive traits, and this pattern already was reported^34^. Another gene responsible for STGD is the *ELOVL4*^35^ gene, however, in the current cohort, no patient had mutations in this gene. On the other hand, pathogenic variants in the *PRPH2* gene, usually known for causing other macular dystrophies^5^, were detected in patients with STGD^36^.

As previously mentioned, the IRDs have striking features such as clinical heterogeneity and significant phenotypic overlap among the different retinopathies^4^. These factors are obstacles to conclusive clinical diagnoses and partially explain the large number of undiagnosed cases. Other facts that hamper definitive diagnoses are the time of patient evaluation; the presence of other confounding characteristics; lack of information about the signs, symptoms, and family history; and often the impossibility of monitoring disease progression. These observations show how challenging the determination of the IRD diagnosis is, especially without genetic testing. Although genetic tests do not guarantee the molecular conclusiveness of all cases, recent technologies such as NGS solve only 55% to 65% of IRD cases^37,38^. Throughout the years, molecular diagnostic methods have been changing. Some initial molecular evaluations in this study had been performed more than a decade previously using the array approach, which detects only known and predetermined variants. Over time, Sanger sequencing, NGS panels, and exomes have been introduced. Moreover, in the late 1990s, about 50 IRD-causing genes were identified; today more than 250 have been associated with retinopathies^5^. Hence, many important genes had not been analyzed in the earlier examinations. Those are some possible reasons for negative and inconclusive cases. In addition, there are technical limitations in NGS analysis such as inaccurate detection of copy number variations, complex genomic rearrangements, low-depth or uncovered regions^39^, genetic aspects such as pathogenic synonymous variants^40^, deep intronic mutations^41^, multigenic inheritance patterns, and gene expression regulators.

The American Academy of Ophthalmology recommends genetic testing, mainly, the most specific tests, when clinical findings suggest a known Mendelian disorder^42,43^. Currently, increasing numbers of molecular tests are orienting clinical practice in retinal dystrophy cases. Some patients had their diagnoses better defined after genetic testing over the previous 20 years, e.g., a few cases diagnosed initially as LCA were reclassified after a molecular test as Senior-Løken, Joubert, or Alström syndromes. The vast majority of changes occurred because the patients were assessed at infancy (birth to 2 years old) and impairment of the other systems could not be detected easily at that time.

Genetic testing is extremely relevant and provides definitive and accurate diagnoses, supports genetic and family counseling, and in the near future will define the appropriate gene therapy for each case^43^. Unfortunately, the above recommendation cannot always be fulfilled. In the current study, more than half of patients did not have access to genetic testing due to its high costs. In Brazil, an NGS panel of about 200 genes for hereditary retinopathy costs three times more than the Brazilian average monthly household income per capita, and the Brazilian public health system does not provide genetic testing for ocular diseases.

An alternative to increase the access to molecular tests is the development of smaller and cheaper gene panels according to the IRD genetic profile of each population. Based on the current results, some types of IRD NGS panels can be developed that focus on the Brazilian population, such as a panel for IRDs with macular impairment (*ABCA4*, *BEST1*, *PROM1*, *PRPH2*, *RS1*) or a panel for childhood dystrophies (*ABCA4*, *AHI1*, *CEP290*, *CLN3*, *CNGB1*, *CRB1*, *CRX*, *GUCY2D*, *IQCB1*, *LCA5*, *LRAT*, *NMNAT1*, *NPHP4*, *PDE6B*, *PROM1*, *PRPH2*, *RDH12*, *RP1*, *RPE65*, *RPGRIP1*, *SPATA7*, *TULP1*). Therefore, other population studies such as this one are important because they provide knowledge about specific populations, increase the diagnostic rates, accurate treatment, genetic counseling, and improve patients’ quality of life.

## Methods

The medical records of 1,246 Brazilian patients with IRDs who were members of 1,159 different families (906 sporadic cases; 161 related patients) were reviewed retrospectively. Of these patients, 559 (from 514 families; 426 sporadic cases; 84 related patients) had undergone at least one genetic test. All patients were assisted at the retinal dystrophy clinic of the Universidade Federal de São Paulo or Instituto de Genética Ocular between January 1998 and February 2018.

Ophthalmologic evaluations were performed in all patients based on visual function tests, visual acuity, color vision tests, visual field, light sensitivity tests, electroretinography, optical coherence tomography, fundus photography, and autofluorescence examinations. Medical and family histories were collected and pedigrees were drawn. The initial clinical diagnosis was based on signs and symptoms, age of onset, and ophthalmologic features. The genetic data were compared with the retinal findings to confirm the initial clinical diagnosis or to reclassify them. All patients underwent genetic counseling.

Molecular genetic data obtained from commercial tests were assessed according to the American College of Medical Genetics and Genomics and the Association for Molecular Pathology guidelines^44^ to determine the pathogenicity of each identified mutation using ophthalmologic database to corroborate the findings. Briefly, the commercial laboratories followed these criteria for variant classification: (1) variants that already have been reported as disease-causing in the literature, (2) variants leading to protein loss-of-function, (3) variants absent in population genetic databases, (4) variants present in other affected patients, and (5) variants that have not been reported in the literature, however, are predicted to be disease-causing by *in silico* analysis.

This study was performed in accordance with the Research Ethics Committee of the Universidade Federal de São Paulo, which approved the study protocol (CEP: 0415/2016). When the DNA samples were collected for molecular tests, all patients and/or their legal guardians provided written informed consent for the use of the personal medical data for scientific purposes and publication. In addition, this study was performed in accordance with the ethical standards of the 1964 Declaration of Helsinki and its subsequent amendments.

## Data Availability

The datasets generated during and/or analysed during the current study are available from the corresponding author on reasonable request.
